# Cross-National Analysis of Bereavement From COVID-19 and Depressive Symptoms Among Older Adults in Europe

**DOI:** 10.1093/geroni/igab046.361

**Published:** 2021-12-17

**Authors:** Haowei Wang, Ashton Verdery, Rachel Margolis, Emily Smith-Greenaway

**Affiliations:** 1 The Pennsylvania State University, University Park, Pennsylvania, United States; 2 University of Western Ontario, London, Ontario, Canada; 3 University of Southern California, Los Angeles, California, United States

## Abstract

The COVID-19 pandemic has left older adults around the globe grieving the sudden death of relatives and friends. We examine if COVID-19 bereavement corresponds with older adults’ depressive symptoms in 27 countries, and test for variation by gender and country context. We analyzed the Survey of Health, Ageing and Retirement in Europe (SHARE) COVID-19 data collected from N=51,383 older adults (age 50–104) living in 27 countries between June-August 2020, of whom 1,363 reported the death of a relative or friend from COVID-19. We estimated pooled-multilevel logistic regression models to examine if COVID-19 bereavement was associated with depressive symptoms and worsening depressive symptoms for older men and women, and we tested whether the national COVID-19 mortality rate in their country had an additive, or multiplicative, influence. COVID-19 bereavement from the death of a relative or friend is associated with significantly higher odds of reporting depressive symptoms, and reporting that these symptoms have recently worsened since the outbreak of COVID-19. Net of personal loss, living in a country with the highest COVID-19 mortality rate corresponds further with women’s depressive symptoms; however, living in the midst of more COVID-19 deaths does not alter the implications of personal loss for depressive symptoms. COVID-19 deaths have lingering mental health implications for surviving older adults. Even as the collective toll of the crisis is apparent, bereaved older adults are in particular need of mental health support.

